# QTL mapping for benzoxazinoid content, preharvest sprouting, α-amylase activity, and leaf rust resistance in rye (*Secale cereale* L.)

**DOI:** 10.1371/journal.pone.0189912

**Published:** 2017-12-21

**Authors:** Paweł Milczarski, Piotr Masojć, Paweł Krajewski, Anna Stochmal, Mariusz Kowalczyk, Mihail Angelov, Valentina Ivanova, Małgorzata Schollenberger, Wojciech Wakuliński, Zofia Banaszak, Katarzyna Banaszak, Monika Rakoczy-Trojanowska

**Affiliations:** 1 West Pomeranian University of Technology, Szczecin, Poland; 2 Institute of Plant Genetics, Polish Academy of Sciences, Poznań, Poland; 3 Institute of Soil Science and Plant Cultivation–State Research Institute, Puławy, Poland; 4 Warsaw University of Life Sciences, Warszawa, Poland; 5 Danko Plant Breeders LTD, KOŚCIAN, Poland; 6 Polish Academy of Sciences Botanical Garden—Centre For Biological Diversity Conservation in Powsin Warszawa, Poland; Institute of Genetics and Developmental Biology Chinese Academy of Sciences, CHINA

## Abstract

Mapping population of recombinant inbred lines (RILs) representing 541 × Ot1-3 cross exhibited wide variations of benzoxazinoid (BX) content in leaves and roots, brown rust resistance, α-amylase activity in the grain, and resistance to preharvest sprouting. QTL mapping of major BX species using a DArT-based map revealed a complex genetic architecture underlying the production of these main secondary metabolites engaged in stress and allelopathy responses. The synthesis of BX in leaves and roots was found to be regulated by different QTL. The QTL for the BX content, rust resistance, α-amylase activity, and preharvest sprouting partially overlapped; this points to their common genetic regulation by a definite subset of genes. Only one QTL for BX located on chromosome 7R coincided with the loci of the *ScBx* genes, which were mapped as two clusters on chromosomes 5RS (*Bx3-Bx5*) and 7R (*Bx1-Bx2*). The QTL common for several BX species, rust resistance, preharvest sprouting, and α-amylase activity are interesting objects for further exploration aimed at developing common markers for these important agronomic traits.

## Introduction

The most abundant secondary metabolites produced in rye leaves and roots are cyclic hydroxamic acids and their derivatives benzoxazinoids (BXs) playing various physiological roles such as defense against pests, allelopathic agents, inhibition of germination, and detoxification [1–4]. Rye contains the highest amounts of these compounds in the grain and green tissues, and there is a wide variation of the BX content among cultivars and breeding materials [4–7]. This points to a possibility for initiating breeding strategies aimed at increasing the BX content in rye cultivars, which then may be treated as a source of allelopathic compounds against many weed species [4]. Although the quantities of BX in rye grain were extensively studied, knowledge about the QTL underlying this variation is very limited. In general, this aspect is poorly recognized. According to the best of our knowledge, there are only two examples of research on QTL controlling the BX content, both concerning corn. In the first of these two studies [8] identified eight QTL stable across different environments and the position of the largest QTL co-localized with a majority of the structural genes of the DIMBOA pathway. Zheng *et al*. [9] using the NAM population located a major quantitative trait locus for a high DIMBOA content (concentrations of 1.5 mM or more) on the short arm of chromosome 4 where the BX gene cluster underlying the biosynthesis pathway of BX up to DIMBOA is mapped. Association of the BX content and SNP within the *ScBx1-ScBx5* genes encoding the enzymes of the BX synthesis pathway was reported by Rakoczy-Trojanowska *et al*. [7]; this suggests that there is a functional allelic variation in these structural loci. SNP in the *Bx* genes were also found to be associated with variations of leaf rust resistance and preharvest sprouting (PHS) within a wide collection of genetically diverse rye materials [10]. This finding raises a question on the possible role of BX production in regulating such important agronomic traits as leaf rust resistance, PHS, and α-amylase activity in rye grain. Association mapping performed using DArT Seq markers revealed associated SNPs on chromosomes 1R (3) 2R (1), and 5R (1) for leaf rust resistance, and on chromosomes 1RL (1), 4RL (1), and 5RL (1) for the α-amylase activity along with numerous SNPs associated with PHS on each rye chromosome except 4R [10].

This paper presents a mapping analysis of *Bx* genes, QTL for BX content, leaf rust resistance, PHS, and α-amylase activity, performed in the 541 × Ot1-3 mapping population of recombinant inbred lines of rye.

## Material and methods

### Plant material

The plant material used in the experiments consisted of 98 recombinant inbred lines and parents for the 541 × Ot1-3 mapping population. This population was genotyped and previously used for the construction of an integrated genetic map of rye [11]. The experiment was conducted at the experimental plots in two growing seasons (2013 and 2014) and two locations: the West Pomeranian University of Technology in Szczecin, Poland (WPUT–N 53°26′51.291″, E 14°31′42.1278″) and Danko Plant Breeders Ltd. in Choryń, Poland (DANKO–N 52°02′19.7″, E 16°46′09.0″). Each RIL was represented by 33 plants in a given season, grown in randomized block design in three replications (11 plants per replicate). Further, observations from four experiments designated as Szczecin 2013, Szczecin 2014, Choryń 2013, and Choryń 2014 were scored.

### Methods

#### Phenotyping

The following traits of RILs and parental lines were phenotyped: brown rust resistance (RR), PHS resistance (PHS-R), and α-amylase activity in grain (AMY).

#### RR

The reaction of RILs to *Puccinia recondita* was verified under field conditions where the source of inoculum was the uredospores of the local population of the fungus, naturally occurring in the environment and spontaneously infecting the plants. No special treatments were applied for enhancing the infection potential of the fungus. Rating was performed at the maximum brown rust epidemics intensity and was based on the average evaluation of all the plants in the plot.

The plant infection level was evaluated according to the following six-degree scale:

0 –resistant, no symptoms on plants
1 –resistant, chlorosis/necrosis, and/or single uredinia sporadically visible on leaves
2 –moderately resistant; single small uredinia regularly present on a majority of plants
3 –moderately susceptible; up to 40% of the leaf surface covered with uredinia
4 –susceptible; up to 70% of the leaf surface covered with uredinia
5 –very susceptible; over 70% of the leaf surface covered with uredinia

#### PHS

Three to six spikes from each RIL and each replicate representing individual plant were harvested at full ripeness and kept at room temperature for 3 days. Then, the spikes were sprayed with water for 5 min each day and kept in a moisture chamber at 20°C for 7 days for stimulated sprouting. Percentage of germinated kernels (the lower the resistance, the higher grain germination) was determined to assess PHS resistance (PHS).

#### AMY

The amount of 1.5 g of kernels from each RIL and each replicate showing no external signs of sprouting were milled into flour. Grain used for the α-amylase assay was stored at room temperature for the first 2 weeks after harvest and then kept at 4°C for the 3 months before the analysis. The α-amylase was extracted in 4 mL of distilled water mixed with 1 g of the flour, equilibrated for 10 min at room temperature, and finally, centrifuged at 8000 × rpm at 4°C for 20 min. The simple method of estimating the level of AMY [12] was used in which the diameters of the α-amylase diffusion circles (in millimeters) in the agarose gel containing starch stained with iodine show a linear relationship with the logarithm of the enzyme activity (in enzyme units per milliliter).

The highest values mean for RR, the lowest infection degree; PHS-R, the highest susceptibility; and AMY, the highest enzymatic activity.

#### Sample preparation and analysis of BX content

Plant samples, the above-ground parts (L) and roots (R), were collected two weeks after the start of the vegetation in the spring, when the plants were in the development stage 21–22 BBCH (20–24 Zadoks development stage), in the experiments performed at Choryń. The tissues after cutting were immediately frozen and lyophilized. Further, 100 mg d.w. of the plant material (L and R) was mixed with diatomaceous earth, placed in stainless steel extraction cells, and extracted with 70% methanol at an operating pressure of 10 MPa and at 40°C by using an accelerated solvent extraction system (ASE 200, Dionex, Sunnyvale, CA). The cells were filled with 250/750 mg of LiChroprep RP-18 (40–60 μm, Merck, Germany) for the roots and the above-ground parts, respectively. After evaporating to dryness under reduced pressure, extracts were reconstituted in 1 mL of methanol containing 0.1% (v/v) acetic acid and stored at −20°C. Prior to the analyzes, the extracts were centrifuged for 20 min at 23,000 × *g* at 4°C.

The quantitative analyzes of the BX content were described in detail by Rakoczy-Trojanowska *et al*. [7].

#### ScBx gene mapping

The sequences of the *ScBx1–ScBx5* genes previously sequenced and characterized in the rye inbred line L318 were used [13]. Sequences of the *ScBx1–ScBx5* genes in parental lines 541 and Ot1-3 were compared; this led to the detection of SNPs, which were used as a 3′ ends of the primers. Alternatively, primers were developed within consensus regions, and restriction enzymes were used for revealing the allelic polymorphisms in SNPs. Sequences of primers, PCR conditions, and restriction enzymes used were presented in S1 Protocol. Polymorphisms among parental lines were initially tested within a group of 16 lines of the 541 × Ot1-3 mapping population to check for an appropriate 1:1 Mendelian segregation. Then, all RILs of the mapping population were genotyped. A reference genetic map consisting mainly of DArT markers was used for mapping both ScBx genes and QTL [11]. Mapping was performed using the JoinMap 4.0 computer program [14].

#### Statistical methods

Observations of the BX content were transformed by log_10_(x+1). The analysis of variance (ANOVA) was performed in the mixed model with years and locations as fixed effects, and lines (RILs) as random effects. Variance components were compared to (3 × std. err.) to assess their magnitude. Broad sense heritability was calculated according to Cullis *et al*. [15]. Correlation coefficient was calculated on average data over years and locations. Computations were performed using Genstat 17 [16].

#### QTL mapping

QTL mapping was performed with the software package WinQTL Cartographer 2.5 [17] using the procedure for composite interval mapping (CIM), with the threshold value of LOD set at a score at 3.0. QTL with an LOD value between 2.5 and 3.0 was considered putative.

## Results

### QTL for benzoxazinoid (BX) content

**Fig 1:**
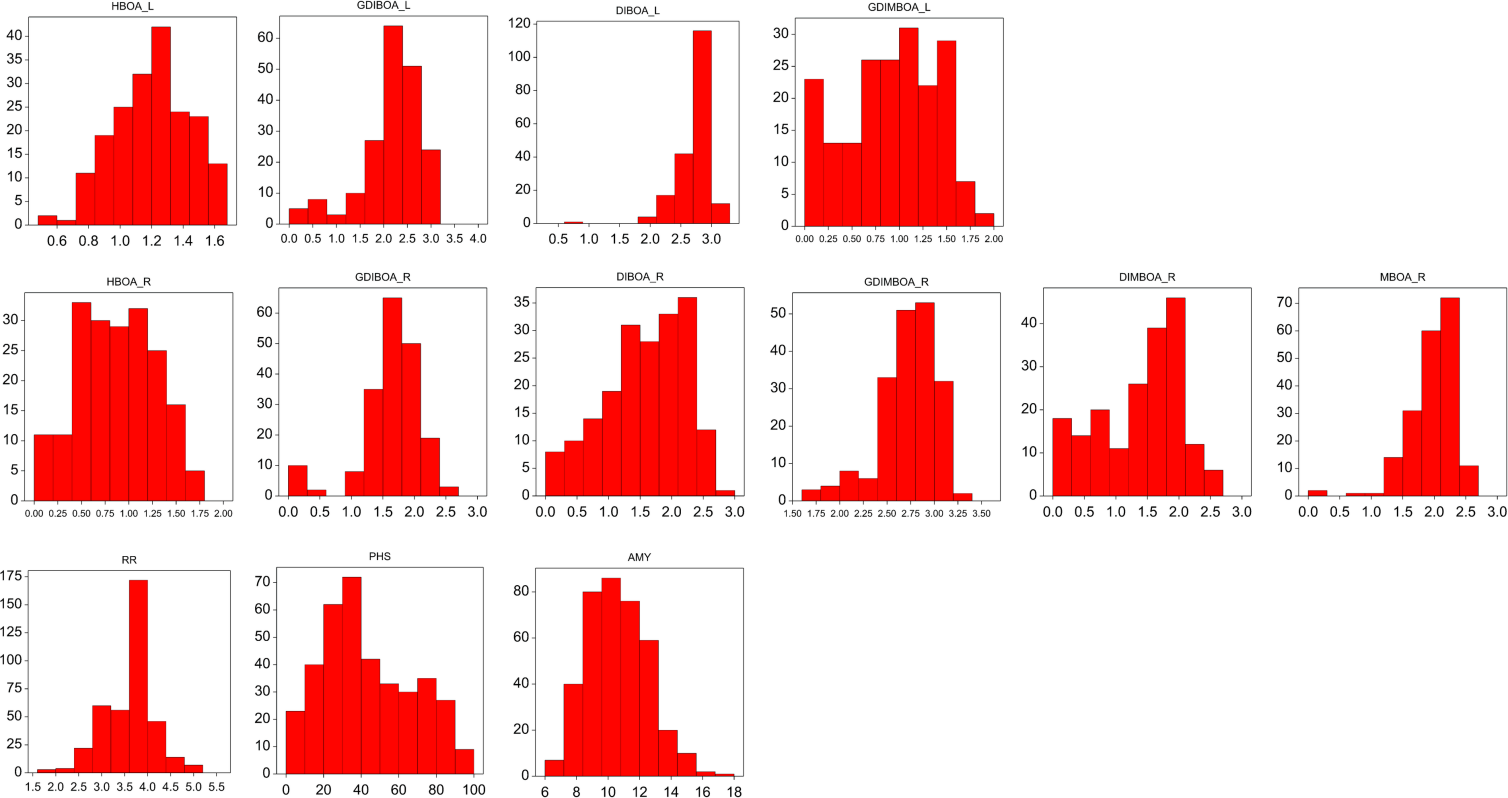
Distribution of variation of BX, RR, PHS and AMY within the 541×Ot1-3 mapping population of RILs after log_10_(x+1) conversion.

**Fig 2:**
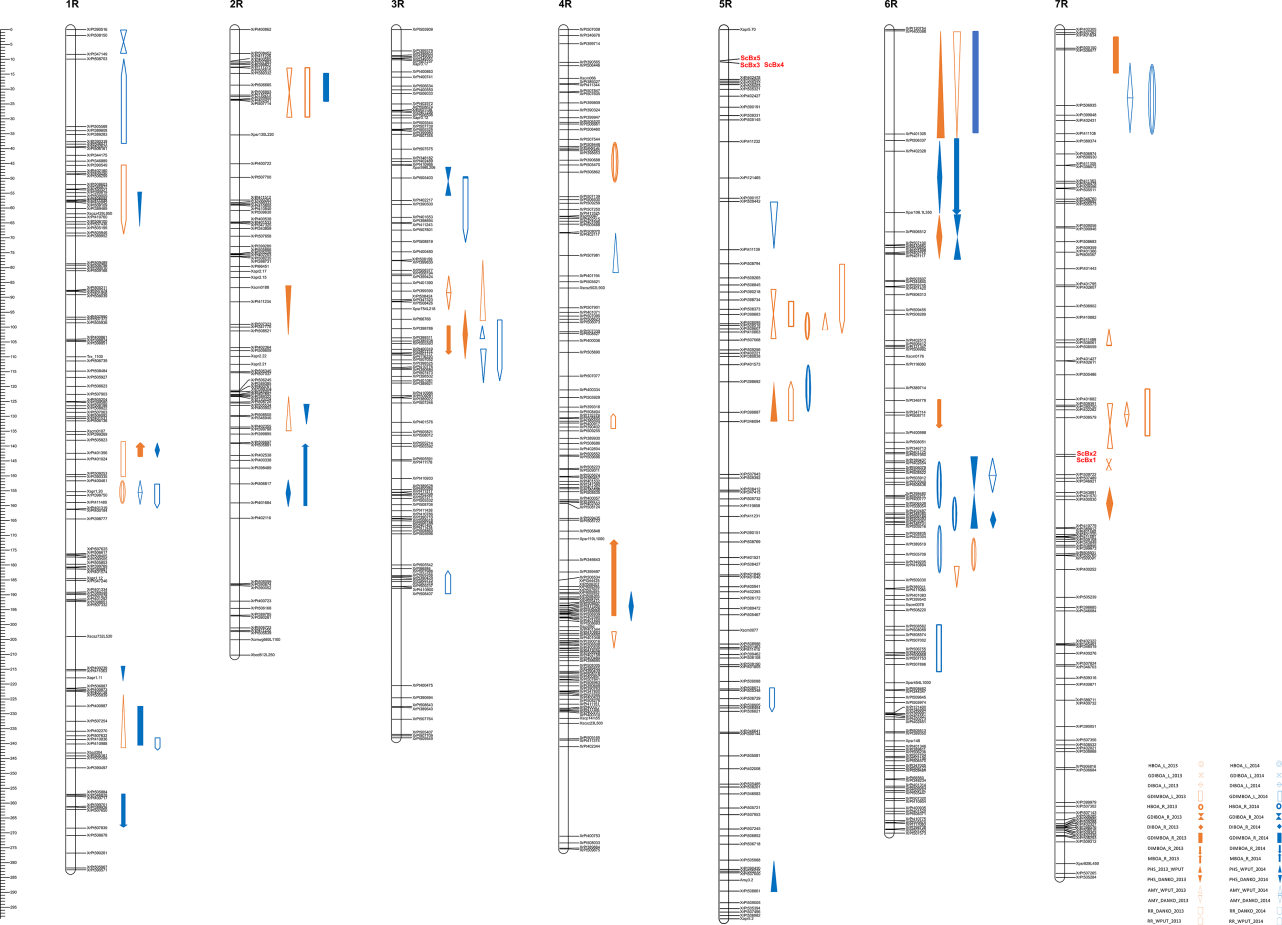
Localization *ScBx1 –ScBx5* genes and QTL of BX, RR, PHS and AMY on the 541×Ot1-3 genetic map.

Differences in BX quantities between the parental lines were low (although statistically significant) and unstable in consecutive years (Table 1). Variation ranges found among RILs were much larger than those found in the parental generation in both years (from about 1.5 to 47 times), which suggests a substantial genetic transgression component in the observed variation. The prevailing BX species in rye leaves were DIBOA and GDIBOA. Lower amounts of GDIMBOA and HBOA were found in this tissue. Roots contained higher amounts of GDIMBOA, MBOA, and GDIBOA, and lower amounts of DIBOA, DIMBOA, and HBOA. Broad sense heritability of the BX species ranged between c. 30% and 40% with two exceptions (16.95% for GDIMBOA_R and 55.36% for MBOA_R, Table 2). But, the substantial lines × years interaction (Table 3) and apparent deviations from the normal distribution lowered the chances of finding stable QTL across different environments (Fig 1). A number of significant QTL for particular BX species were found (Tables 4 and 5, Fig 2). Depending on a year of study, 2 (2014) to 3 (2013) QTL were detected for HBOA in leaves, with one common on chromosome 6RL and three separate on chromosomes 1R, 4RS, and 7RS. QTL for HBOA in roots were found on chromosome 5R in 2013 and on chromosomes 5R (1) and 6R (2) in 2014. The QTL mapping of the GDIBOA amount in leaves revealed 1 QTL in 2014 and 4 QTL in 2013, located on the distal part of chromosome 1RS (1); distal part of chromosomes 2RS (1), 5R (1), and 7RL (2) coinciding with the locus for the *ScBx1-2* genes. No QTL for GDIBOA in roots were found in 2013, but in 2014, there were 3 detected QTL on chromosomes 3RS, 6RS, and 6RL. QTL for DIBOA in leaves were dispersed on chromosomes 3R and 7RL (adjacent to the *ScBx1* locus) in 2013, whereas in 2014, they were detected on chromosomes 1R, 6R, and 7R. The QTL for DIBOA in roots was found on chromosomes 3R, 6RS, and 7RL in 2013 and on chromosomes 1RL, 2RL, 4RL, and 6R (2) in 2014. Closely linked but not overlapping QTL on chromosomes 1R, 3R, and 6R were detected for DIBOA from both leaves and roots. The QTL for GDIMBOA from leaves were mapped on chromosomes 1R, 2RS, 5RS, and 7R in 2013 and only on 6R in 2014. The distribution of GDIMBOA QTL in leaves partially overlapped with that in roots where the QTL were located on chromosomes 1R, 2RS (coinciding QTL found in leaves and roots), and 6RS in 2014 and on chromosome 7R (2013) near the *ScBx1* locus. DIMBOA QTL were detected only in roots. They were located on chromosomes 3R and 6R in 2013 and on chromosomes 1RL and 6RS in 2014. MBOA QTL were not detected in leaves. MBOA QTL in roots were represented in 2013 by two loci on chromosomes 1R and 4RL and by one locus on chromosome 2RL in 2014.

**Table 1:** Characteristics of BX contents (μg/g d.w.) in leaves (L) and roots (R).

BX	Year	Parents					RILs					
		P1 OT1-3	Std. err.	P2 541	Std. err.	P1-P2	No. of lines	Mean	Std. error of mean	Min	Max	C.V. [%]
1. HBOA_L	2013	1.068	0.054	1.262	0.141	-0.194*	96	1.218	0.029	0.543	1.646	23.10
	2014	1.198	0.062	1.032	0.047	0.166*	96	1.194	0.018	0.752	1.611	15.10
2. GDIBOA_L	2013	2.459	0.018	1.882	0.215	0.577*	96	1.719	0.062	0	2.383	35.20
	2014	2.499	0.009	2.62	0.066	-0.121*	96	2.616	0.024	2.013	3.084	9.10
3. DIBOA_L	2013	2.774	0.036	2.725	0.023	0.049	96	2.618	0.034	0.725	3.049	12.60
	2014	2.886	0.037	2.746	0.070	0.140*	96	2.829	0.014	2.498	3.104	4.80
4. GDIMBOA_L	2013	1.756	0.096	0.504	0.275	1.252*	96	0.967	0.058	0	1.837	59.00
	2014	0.785	0.031	1.298	0.070	-0.513*	96	0.853	0.040	0	1.549	45.40
5. HBOA_R	2013	1.279	0.032	1.177	0.043	0.102	96	1.199	0.024	0.679	1.758	19.60
	2014	0.788	0.009	0.261	0.043	0.527*	96	0.556	0.026	0	1.244	45.40
6. GDIBOA_R	2013	1.950	0.045	1.559	0.106	0.391*	96	1.494	0.06	0	2.23	39.40
	2014	1.878	0.040	1.444	0.152	0.434*	96	1.761	0.036	1.010	2.564	20.30
7. DIBOA_R	2013	2.361	0.071	2.467	0.026	-0.106	96	2.050	0.035	1.014	2.773	16.50
	2014	1.200	0.083	0.912	0.298	0.288	96	1.115	0.053	0	2.453	46.30
8. GDIMBOA_R	2013	2.948	0.035	2.811	0.032	0.137*	96	2.674	0.03	1.726	3.123	11.10
	2014	3.017	0.062	2.839	0.107	0.178*	96	2.781	0.032	1.628	3.270	11.20
9. DIMBOA_R	2013	2.123	0.083	2.062	0.050	0.061	96	1.727	0.048	0	2.635	27.50
	2014	1.217	0.109	1.903	0.072	-0.686*	96	1.056	0.068	0	2.531	63.50
10. MBOA_R	2013	2.347	0.073	2.528	0.029	-0.181*	96	2.126	0.025	1.441	2.673	11.30
	2014	2.196	0.063	1.985	0.053	0.211*	96	1.826	0.042	0	2.333	22.50

*—difference between parents significant according to the LSD test at P < 0.05

**Table 2:** Characteristics of rust resistance (RR), preharvest sprouting (PHS) and alpha-amylase activity in rye grain (AMY).

Trait	Year	Location	Parents					RILs					
			P1 OT1-3	Std. err.	P2 541	Std. err.	P1-P2	No. of lines	Mean	Std. err.	Min	Max	C.V. (%)
11. RR	2013	Choryn	4.00	0.58	4.00	0.58	0	96	4.18	0.04	3.33	5.00	9.53
		Szczecin	3.33	0.33	4.00	0.00	-0.67	96	3.58	0.03	3.00	4.33	9.02
	2014	Choryn	4.00	0.00	4.33	0.33	-0.33	96	3.88	0.04	3.00	4.67	9.42
		Szczecin	3.33	0.33	3.33	0.88	0	96	2.97	0.04	2.00	4.00	12.71
12. PHS	2013	Choryn	41.11	9.81	71.02	5.58	-29.91*	93	33.41	1.54	0	68.26	44.53
		Szczecin	18.02	2.24	89.92	3.80	-71.90*	96	49.14	2.97	4.43	95.08	59.25
	2014	Choryn	52.96	10.65	52.97	3.11	-0.01	91	35.49	1.70	2.63	82.14	45.61
		Szczecin	50.46	10.16	82.58	0.76	-32.12*	93	55.96	2.55	4.55	100.00	44.01
13. AMY	2013	Choryn	16.83	1.09	10.33	0.33	6.50*	94	11.27	0.16	8.00	15.33	14.18
		Szczecin	16.67	0.44	13.33	0.60	3.34*	96	10.95	0.20	7.33	15.67	17.93
	2014	Choryn	11.33	1.60	9.00	0.00	2.33	96	8.97	0.12	6.00	12.00	12.96
		Szczecin	16.67	0.93	12.67	0.88	4.00*	95	11.29	0.18	7.00	17.00	15.54

*—difference between parents significant according to the LSD test at P < 0.05

**Table 3:** ANOVA results for BX quantities in rye tissues.

Trait	P-value for Years	Variance component for Lines	Std. error	Variance component for interaction Lines x Years*	Std. error	Heritability (%)
HBOA_L	0.430	0.011	0.006	0.040*	0.006	34.41
GDIBOA_L	< 0.001	0.055	0.022	0.143*	0.022	41.33
DIBOA_L	< 0.001	0.017	0.007	0.041*	0.007	41.73
GDIMBOA_L	0.051	0.075	0.025	0.145*	0.023	47.95
HBOA_R	< 0.001	0.013	0.006	0.042*	0.007	35.82
GDIBOA_R	< 0.001	0	-	0.235*	0.035	-
DIBOA_R	< 0.001	0.029	0.019	0.133*	0.023	26.84
GDIMBOA_R	0.011	0.008	0.009	0.078*	0.012	16.95
DIMBOA_R	< 0.001	0	-	0.322*	0.049	-
MBOA_R	< 0.001	0.043*	0.012	0.063*	0.010	55.36

*—significance of variance components assessed approximately by comparison with (3 x std. error)

**Table 4:** Characteristics of QTL detected in 2013.

Trait	QTL	Chromosome	Marker interval	QTL position (cM)	LOD	Additive effect	R^2^ (%)
HBOA_L	HBOA_AG/13_1	1R	XrPt390335—XrPt411480	155.3	2.8	-3.19	6.7
	HBOA_AG/13_2	4R	XrPt398853—XrPt506862	45.5	3.9	-4.11	10.7
	HBOA_AG/13_3	6R	XrPt508835—XrPt410804	176.4	4.5	-4.40	14.4
GDIBOA_L	GDIBOA_AG/13_1	2R	XrPt505615—XrPt507714	25.8	8.6	41.42	34.0
	GDIBOA_AG/13_2	5R	XrPt398734—XrPt507068	99.5	2.9	-20.28	7.6
	GDIBOA_AG/13_3	7R	XrPt508579 –ScBx2	128.2	5.9	34.46	16.5
	GDIBOA_AG/13_4*	7R	ScBx1 –XrPt509722	139.8	2.6	21.05	8.2
DIBOA_L	DIBOA_AG/13_1	3R	XrPt399424—Xpsr754L218	91.3	3.7	-97.35	12.9
	DIBOA_AG/13_2	7R	XrPt508579 –ScBx2	128.2	3.5	87.84	12.1
GDIMBOA_L	GDIMBOA_AG/13_1	1R	XrPt399269—XrPt509253	142.4	3.4	5.46	10.5
	GDIMBOA_AG/13_2	2R	XrPt505615—XrPt507714	23.6	4.5	6.12	14.2
	GDIMBOA_AG/13_3*	5R	XrPt398734—XrPt509607	98.8	2.6	-4.64	7.8
	GDIMBOA_AG/13_4	7R	XrPt508579 –ScBx2	128.2	3.9	5.82	12.3
HBOA_R	HBOA_R/13_1	5R	XrPt508373—XrPt507068	98.9	3.2	-4.77	10.8
DIBOA_R	DIBOA_R/13_1	3R	XrPt66766—XrPt116220	104.7	3.4	-40.95	9.7
	DIBOA_R/13_2	6R	Xpsr106.1L350—XrPt401117	72.6	2.9	-37.88	8.3
	DIBOA_R/13_3	7R	XrPt507302—XrPt508293	158.3	4.5	-46.37	13.2
GDIMBOA_R	GDIMBOA_R/13_1*	7R	XrPt401834—XrPt506935	9	2.6	-104.20	14.7
DIMBOA_R	DIMBOA_R/13_1	3R	XrPt398766—XrPt507717	109	3.3	-27.41	10.0
	DIMBOA_R/13_2	6R	XrPt347114—XrPt508051	129.6	3.6	28.46	11.1
MBOA_R	MBOA_R/13_1	1R	XrPt399269—XrPt401024	140	3.2	32.58	10.7
	MBOA_R/13_2	4R	Xpsr119L1000—XrPt505236	187.2	5.1	-44.84	17.0
RR_DANKO	RR_DANKO/13_1	1R	XrPt390549—XrPt505946	54.8	4.9	0.16	15.6
	RR_DANKO/13_2	5R	XrPt508794—XrPt4100863	94	4.2	-0.15	13.3
RR_WPUT	RR_WPUT/13_1	4R	XrPt508495—XrPt400917	130.5	3.1	0.12	10.5
	RR_WPUT/13_2	5R	XrPt401573—XrPt346094	135.8	4.3	-0.17	25.5
PHS_DANKO	PHS_DANKO/13_1	2R	Xscm0188—XrPt508521	97.5	5.0	-7.76	22.2
PHS_WPUT	PHS_WPUT/13_1	5R	XrPt401573—XrPt346094	123.4	4.0	-12.63	17.7
	PHS_WPUT/13_2	6R	XrPt100398 -XrPt401305	21.4	3.0	23.44	45.5
	PHS_WPUT/13_3	7R	XrPt506902—XrPt508061	102.1	3.8	-10.92	12.2
AMY_WPUT	AMY_WPUT/13_1	1R	XrPt400138—Xbcd304	235.8	5.0	-0.77	13.7
	AMY_WPUT/13_2	2R	XrPt120224—XrPt399788	125.2	3.6	0.69	10.3
	AMY_WPUT/13_3	3R	XrPt399830—XrPt398766	90.8	5.3	-0.78	14.5
	AMY_WPUT/13_4*	5R	XrPt508373—XrPt508518	98.9	2.7	-0.54	6.9
AMY_DANKO	AMY_DANKO/13_1*	4R	XrPt509036—XrPt506651	206.9	2.6	-0.70	8.7
	AMY_DANKO/13_2	6R	XrPt100398 -XrPt401305	1.4	7.9	1.20	38.8
	AMY_DANKO/13_3	6R	XrPt410804—XrPt411086	187.8	2.9	-0.80	7.6

**Table 5:** Characteristics of QTL detected in 2014.

Trait	QTL	Chromosome	Marker interval	QTL position (cM)	LOD	Additive effect	R^2^ (%)
HBOA_L	HBOA_AG/14_1	6R	XrPt346701-XrPt410804	170.3	4.9	-2.87	16.1
	HBOA_AG/14_2	7R	XrPt505150-XrPt402431	15	3.5	2.30	9.9
GDIBOA_L	GDIBOA_AG/14_1	1R	XrPt390516-XrPt347149	0	5.2	111.87	19.2
DIBOA_L	DIBOA_AG/14_1	1R	XrPt509253-XrPt411480	154.7	3.1	-65.95	9.3
	DIBOA_AG/14_2	6R	XrPt401725-XrPt508436	147.5	3.4	-69.48	9.9
	DIBOA_AG/14_3	7R	XrPt505150-XrPt402431	16	4.5	83.18	13.7
GDIMBOA_L	GDIMBOA_AG/14_1	6R	XrPt508562-Xpsr454L1000	212.4	4.4	3.62	15.3
HBOA_R	HBOA_R/14_1	5R	XrPt389838-XrPt399887	122.4	3.5	0.95	12.9
	HBOA_R/14_2	6R	XrPt402654-XrPt508054	157	5.3	-1.12	17.5
	HBOA_R/14_3*	6R	XrPt509216-XrPt410804	165.3	2.6	-0.81	9.3
GDIBOA_R	GDIBOA_R/14_1*	3R	XrPt410966-XrPt505403	49.9	2.7	19.83	7.3
	GDIBOA_R/14_2	6R	Xpsr106.1L350-XrPt401117	72.4	3.7	-24.61	10.7
	GDIBOA_R/14_3	6R	XrPt507940-XrPt506539	150.8	5.0	-27.97	14.6
DIBOA_R	DIBOA_R/14_1	1R	XrPt399269-XrPt401024	138	2.8	-8.42	5.2
	DIBOA_R/14_2	2R	XrPt398489-XrPt402116	159.1	3.6	-10.39	7.9
	DIBOA_R/14_3	4R	XrPt508265-XrPt402590	195.4	3.0	-12.16	5.9
	DIBOA_R/14_4*	6R	XrPt401305-Xpsr106.1L350	40.9	2.7	-8.79	5.7
	DIBOA_R/14_5	6R	XrPt398480-XrPt400777	157	3.1	-12.07	10.4
GDIMBOA_R	GDIMBOA_R/14_1	1R	XrPt505839-XrPt410988	233.5	3.6	157.57	12.1
	GDIMBOA_R/14_2	2R	XrPt389332-XrPt402421	23.4	3.8	147.16	11.7
	GDIMBOA_R/14_3*	6R	XrPt100398-XrPt401305	19.4	2.7	-274.70	39.3
DIMBOA_R	DIMBOA_R/14_1	1R	XrPt389926-XrPt508678	268.1	3.3	30.01	11.6
	DIMBOA_R/14_2	6R	XrPt506337-Xpsr106.1L350	40.9	3.1	-15.70	9.9
MBOA_R	MBOA_R/14_1	2R	XrPt505891-XrPt401684	152.7	4.1	-22.42	13.5
RR_DANKO	RR_DANKO/14_1	1R	XrPt400461-XrPt411480	154.7	3.9	0.13	12.1
	RR_DANKO/14_2	1R	XrPt507632-Xbcd304	240.1	3.0	-0.11	8.0
	RR_DANKO/14_3	3R	XrPt505403-XrPt508819	57.4	3.3	-0.11	8.7
	RR_DANKO/14_4	3R	XrPt66766-XrPt390684	105.5	4.8	0.14	13.3
	RR_DANKO/14_5*	5R	XrPt508071-XrPt506821	224.9	2.6	-0.10	7.0
RR_WPUT	RR_WPUT/14_1	1R	XrPt508703-XrPt344175	39.4	3.0	0.12	10.0
	RR_WPUT/14_2	3R	XrPt507316-XrPt508407	187.3	3.0	-0.12	10.4
PHS_DANKO	PHS_DANKO/14_1	1R	XrPt390321-XrPt506100	54.8	2.9	-5.29	8.9
	PHS_DANKO/14_2	1R	XrPt400239-XrPt506887	217.9	2.8	-5.29	8.7
PHS_WPUT	PHS_WPUT/14_1	5R	XrPt506718-XrPt508861	286.9	4.0	-10.22	14.5
AMY_DANKO	AMY_DANKO/14_1	2R	XrPt505534-XrPt346940	128.3	2.9	0.38	9.9
	AMY_DANKO/14_2	3R	XrPt400319-XrPt401081	109	4.0	-0.42	12.2
	AMY_DANKO/14_3	5R	XrPt390157-XrPt508794	70.8	3.0	-0.47	15.5
AMY_WPUT	AMY_WPUT/14_1	3R	XrPt398786-XrPt505593	103.6	3.4	-0.74	11.3
	AMY_WPUT/14_2	4R	XrPt509488-XrPt401194	68.2	3.6	-0.78	12.6

The R^2^ value for most of the QTL for BX was close to 10%. The greatest impact on the BX content in leaves was exerted by GDIBOA_L/13_1 (R^2^ = 34%) on chromosome 2R (2013), and in roots, by GDIMBOA_R/14_3 (R^2^ = 39.3%) on chromosome 6R (2014). QTL were significant only in one year of the study (with one exception on 6RL), which illustrates their instability due to the significant environmental component of variation including lines versus years relationship.

Except for the QTL on the distal part of chromosome 2RS, there were no coinciding QTL for the same BX species in leaves and roots, which strongly supports the hypothesis of the differences in the regulation of BX production in these two plant tissues. In contrast, there are several coinciding QTL for different BX species like those for leaf GDIBOA, GDIMBOA, and HBOA on 5RS; leaf GDIBOA and GDIMBOA on 2RS; and leaf GDIBOA, GDIMBOA, and DIBOA on 7R. Similarly, coinciding QTL for different types of BX in roots were found on chromosomes 2RL (DIBOA and MBOA), 3R and 6RS (DIMBOA and DIBOA), 4RL (MBOA and DIBOA), and 6RS (GDIBOA and DIBOA). They may represent pleiotropic genes regulating the production of more than one BX species or clusters of tightly linked genes with specificity for a given species of BX. Coinciding QTL for BX in both leaves and roots may constitute the genetic background for the significant correlation of their amounts detected in this study (Table 6).

**Table 6:** Significant correlation coefficients between studied traits (P < 0.01).

	HBOA_L	GDIBOA_L	DIBOA_L	GDIMBOA_L	HBOA_R	GDIBOA_R	DIBOA_R	GDIMBOA_R	DIMBOA_R	MBOA_R	RR	PHS	AMY
HBOA_L	1.00												
GDIBOA_L	0.52	1.00											
DIBOA_L	0.49	0.46	1.00										
GDIMBOA_L	0.29	0.77		1.00									
HBOA_R	0.47				1.00								
GDIBOA_R	0.38	0.30			0.65	1.00							
DIBOA_R				-0.25	0.75	0.61	1.00						
GDIMBOA_R				0.33		0.40		1.00					
DIMBOA_R		0.31		0.47		0.31		0.80	1.00				
MBOA_R		-0.33						0.39		1.00			
RR											1.00		
PHS												1.00	
AMY													1.00

Structural loci encoding enzymes of the BX biosynthesis pathway were mapped on the distal part of chromosome 5RS (a cluster of the *ScBx3*, *ScBx4*, and *ScBx5* genes) and on the central part of the 7R chromosome (a cluster of the *ScBx1* and *ScBx2* genes) (Fig 2). The QTL for BX in leaves and roots did not coincide with these structural genes except for the QTL for GDIMBOA in roots, which was mapped near the *ScBx1* and *ScBx2* gene cluster on chromosome 7R.

### QTL for resistance to preharvest sprouting (PHS)

In each experiment with one exception (Choryń 2014), differences between parental lines were substantial, showing that line Ot1-3 represents resistant and line 541 susceptible phenotypes (Table 2). The RIL variation ranges were large in each experiment overcoming those of parental lines, which led to high coefficients of variation. But, because of a very high line × location and line × year interactions, the heritability of the trait calculated for the whole experiment was very low (2.18%) (Table 7). Only four QTL for PHS were detected in 2013 and three others in 2014 (Tables 4 and 5, Fig 2). The highest determination coefficient has PHS_WPUT/13_2 QTL from the distal part of chromosome 6RS. The QTL for PHS were distributed on 1RS (1), 1RL (1), 2R (2), 5R (2), and 6RS (1).

**Table 7:** ANOVA results for traits.

Trait	P-value for Years (Y)	P-value for Locations (P)	P-value for interaction Y x P	Variance component for Lines (L)	Std. error	Variance component for interaction L x Y	Std. error	Variance component for interaction L x P	Std. error	Heritability (%)
RR	< 0.001	< 0.001	< 0.001	0.026	0.009	0.008	0.008	0	-	52.14
PHS	0.005	< 0.001	0.175	4.40	38.50	67.47	18.88	264.38	46.99	2.18
AMY	< 0.001	< 0.001	< 0.001	1.43	0.30	0.20	0.08	0.55	0.132	71.85

### QTL for α-amylase activity (AMY)

The grains of parental lines exhibited large differences in the α-amylase activity, and a large variation range was detected among RILs in each experiment (Table 2).

The environmental component of variation due to the line × location interaction was significant (Table 7). In contrast to the other traits, the heritability of the α-amylase activity proved to be very high (71.85%), and its distribution was close to normal (Fig 1). Among the seven QTL detected in 2013, the one on chromosome 6R was the most efficient (R^2^ = 38.8%) (Table 4, Fig 2). Five QTL detected in 2014 had similar low determination coefficients (c. 10%) (Table 5, Fig 2). QTL were found on distal parts of chromosome 1RL, on the proximal part of chromosome 2RL (present in two years), on the proximal part of chromosomes 2RL and 3R in three adjacent positions), and on chromosomes 4RS, 4RL, 5RS, 6RS, 6RL, and 7RS.

### QTL for leaf rust resistance (RR)

Parental lines did not differ significantly with respect to rust resistance (Table 7). But, the variation ranges among RILs were substantial because of transgression effects, which assured that the genetic variation in a mapping population although not high (CV close to 10%) was sufficient for successful QTL mapping. In addition, the results of ANOVA showed that the environmental component of variation was low and the heritability was high (Table 4).

Chromosome 1R carried four QTL for leaf rust resistance. They were distributed on the distal part of the 1RS arm (2) and on the 1RL arm (2) (Tables 4 and 5, Fig 2). Three QTL for rust resistance were detected on chromosome 3R: the first on the short arm, the second in the centromeric region 3Rc, and the third on the long arm. Chromosome 4R contained one Lr QTL on the 4Rc. Two other QTL for rust resistance were in the 5Rc region. A majority of the RR QTL determined the trait variation in c. 10%, while the RR QTL on chromosome 5R (RR_WPUT/13_2) had a much higher impact (25.5%). Each of the RR QTL was detected only in one year and in one location, which shows that their effectiveness was highly dependent on environmental changes.

### Coinciding QTLs for different traits

The most interesting was the coinciding of QTL for rust resistance affecting leaves and the α-amylase activity in rye grain. Such cases were found on chromosomes 1RL and 3R (Fig 2), but they did not result in a significant correlation between these traits (Table 6). The two coinciding QTL for PHS and rust resistance were detected on chromosomes 1RS and 5R (Tables 4 and 5, Fig 2). Except for the distal region on chromosome 1RL, the proximal region of 2RL, and the distal region of 6RS, there were no genomic locations with coinciding PHS and AMY QTL, which may partially confirm the lack of correlation between these two traits (Table 6).

The QTL for BX in leaves or roots were found in several locations accompanied by AMY QTL as on chromosomes 1RL, 3R, 5R, 6RS, and 6RL. The most apparent coincidence for PHS and GDIMBOA QTL from roots was on the distal part of chromosome 6RS. A common position of QTL for the HBOA content in roots and the PHS QTL was also detected on chromosome 5R. The detected similarities between BX and AMY or PHS QTL distribution did not result in a significant correlation between these traits (Table 6). Coinciding QTL for different traits raise a question on the pleiotropic effects of the underlying genes. They also provide strong support for the validity of the QTL detection.

## Discussion

Results of mapping the *ScBx* genes were in agreement with the earlier reports on their distribution in the contigs of the rye BAC library [13]. The *ScBx3*, *ScBx4*, and *ScBx5* genes were mapped as a one gene cluster on the distal part of chromosome 5RS and *ScBx1* with the *ScBx2* gene as another cluster on chromosome 7R. This result clearly shows that both groups of genes are not linked to other BX genes: *ScGT* encoding UDP-glucosyltransferase and *ScGlu* encoding beta-glucosidase which were shown to be located on chromosomes 4R and 2R, respectively [18]. The map positions of the *ScBx1-5* cluster on 5RS and the *ScBx1-2* cluster on 7R, determined for the first time in this study, confirm the earlier results attributing the *Bx* genes to rye chromosomes [18,19]. The map position of *Bx* genes in the rye genome is thus not syntenious to that found in corn where they are all clustered on the distal part of the short arm of chromosome 4 [20]. The two clusters of the BX genes were also assigned to chromosomes in wheat: *Bx1* and *Bx*2 to chromosome group 4 and *Bx3*, *Bx4*, and *Bx5* to the short arm of the chromosomes of homoeologous group 5 [18]. Since a segment of chromosome 4L is located on rye chromosome 7RS because of ancient translocation [21], it may be inferred that the *Bx1-2* cluster from wheat chromosome 4DL and that from rye chromosome 7R are located synteniously.

The studied complex QTL system for BX in the rye genome constitutes a network of regulatory genes affecting the expression of the structural *Bx* genes encoding the enzymes of the BX biosynthesis pathway. The *Bx* genes located in two clusters on chromosomes 5RS and 7R have no or a slight effect on the variation of BX production. Only one QTL for GDIBOA_L with a determination coefficient of 16.5% and additive value of 34.5 coincided in one year with the *Bx1-2* genes on chromosome 7R. A similar situation was found for the α-amylase activity in rye where none of the QTL detected coincided with the structural genes encoding the α-amylase isozymes playing a major role in the catabolism of the endosperm starch [22,23]. Allelic forms of the *Bx* structural genes have little effect on their expression. Instead, a high level of genetic variation in regulatory genes enables a fast and specific adjustment of enzyme synthesis in response to environmental conditions. Another observation supporting this hypothesis is that of the completely different sets of QTL regulating the production of a particular BX species in roots and leaves. These organ-specific regulatory networks probably reflect different defensive mechanisms against biotic and abiotic stresses acting upon roots and leaves or may result from the activation of different members of the multigene families in each tissue. A second possible explanation of the apparently small impact of polymorphisms in the *Bx* structural loci on the BX content, found in this study, is the lack of functional polymorphism in the *Bx* genes within the mapping population. This hypothesis is supported by earlier results of association mapping, which showed the presence of SNPs in the *Bx* genes associated with the BX content within a wide range of rye genotypes [7]. Similarly, the results reported by [8] and [9] support the significant role of polymorphisms within the *Bx1* sequences in controlling the BX content in corn.

Results of the QTL mapping revealed that although the heritability of the BX content in rye is promising for breeding purposes, individual QTL are not stable across environments and years. This is a common conclusion from many QTL studies based on the whole mapping populations where phenotypic differences between lines are most often small and prone to environmental factors. This problem is overcome in bidirectional selective genotyping (BSG) studies where only extreme phenotypes, derived through divergent selection and much more independent of environmental changes, are analyzed [22–24]. These studies show that the individual effects of QTL are usually low, but their impact on the trait due to their interactions with other QTL may be substantial.

The complex genetic architecture of BX in rye makes it difficult to develop an efficient strategy for increasing the level of these important metabolites in both leaves and roots. From the QTLs for GDIBOA in leaves, the most promising for selection purposes might be those on chromosomes 1RS, 2RS, and 7R (coinciding with the *ScBx1* gene). Both QTL for DIBOA in leaves on chromosome 7R seem to also be valuable for such selection. Roots can be enriched in BX by applying the QTL for GDIMBOA on chromosomes 1R, 2R, 6RS, and 7R.

The set of QTL for PHS revealed in this study constitutes only a part of the genetic architecture detected in the 541 × Ot1-3 intercross in earlier studies [22,25,26]. The QTL on chromosomes 1RL, 2R, 3R, 5R (3), and 6RS are located on the dense DArT map of the RIL population applied in this study in similar positions as those found on the F_2_ map consisting mainly of the RAPD and SSR markers [27] and agree with the results of association mapping performed within a wide range of rye genotypes [10]. The present experiment however did not identify the QTL for PHS on chromosomes 3RS, 6RL, and 7RS detected earlier by using both QTL mapping and BSG analysis. A proteomic analysis of lines representing the resistant and susceptible lines of the 541 × Ot1-3 cross showed that many identified proteins represented defensive functions against microbial and insect pests [28,29]. This should not be surprising as only the intact grain surface without any destructive effects of pets’ activities can resist water infiltration, which directly induces sprouting and the α-amylase production. This observation leads to the hypothesis about the possible role of BX in the resistance to PHS, which can be additionally supported by data about the inhibitory effect of BX on the germination ability [4]. The results of this study suggest that the coinciding positions of QTL for PHS and BX may reflect the common regulation of BX production and PHS in rye grain.

One of the first processes connected with the water uptake by the grain is the increase in the α-amylase activity in the endosperm. Therefore, all factors reducing the water permeability of the grain surface, including resistance against microbial infections and pest infestation should hinder the α-amylase production in aleurone cells. BXs are among metabolites that play a defensive role, and their relationship with the α-amylase activity is an important question. The negative effect of BX on a plant’s α-amylase activity was reported by Kato-Noguchi *et al*. [30]. As shown in this paper, several QTL for AMY coincide with those for BX. This observation opens up an opportunity for their common influence on both traits in the rye grain. Future studies should verify this hypothesis, which may play a positive role in finding valuable genes for rye breeding.

The α-amylase activity in the rye grain is a very variable trait, sensitive to environmental conditions, mainly the precipitation and temperature profiles during the period of grain maturation. Levels of this enzyme in the maturing grain were found to be not correlated with its predisposition to PHS [10,31]. Further, results reported in this paper are in agreement with the QTL mapping performed earlier within the 541×Ot1-3 mapping population [23,26,32]. The nine positions of QTL for AMY found here represent regions similar to those detected in previous reports, including an association mapping study [10]. The AMY and PHS QTL occupy similar positions in three chromosomal regions. This finding to some extent confirms earlier results showing that the genetic architecture of these traits is partially overlapping on chromosomes 1RL, 2R, 3R, and 6RL [23,26,33].

Despite several genetic, physiological, and breeding-directed studies, leaf rust caused by *Puccinia recondita f*. sp. *secalis* still constitutes a big challenge for rye breeders [34–36]. The SNPs detected for leaf rust resistance using association mapping on chromosome 1R [10] are in a region of RR QTL found in this study. But, no QTL on chromosome 2RS and 5RL, corresponding to the SNPs detected through association mapping, were found. RR QTL were also not found within regions of chromosomes 6RL and 7RL, where the *Pr1* and *Pr2* genes for leaf rust resistance were mapped [36]. QTL for rust resistance coincide in a majority of cases with the BX QTL and the QTL for the α-amylase activity, along with those for PHS on chromosome 5R. This finding is very important for the possibility of selecting molecular markers aimed at improving AMY, PHS, BX content, and rust resistance in rye. Such markers should be searched for in chromosome regions with coinciding QTL.

The physiological aspect of the common positions of QTL for defensive traits (BX content and rust resistance) and water stress-dependent traits such as PHS and AMY is very interesting. Further, the synthesis of the BX species as defensive metabolites is probably a part of a wider mechanism of mobilizing all necessary plant resources to eliminate the negative aspects of biotic and/or abiotic stresses.

## Supporting information

S1 Protocol Sequences of primers, PCR conditions, and restriction enzymes used for revealing the allelic polymorphisms in SNPs of *ScBx*1-5. (DOCX)

## References

[1] Niemeyer HM. Hydroxamic acids (4-hydroxy-1,4-benzoxazin-3-ones) defense chemicals in the Gramineae. Phytochemistry. 1988;27: 3349–3358.

[2] Pérez FJ, Ormeño-Nuñez J. Difference in hydroxamic acid content in roots and root exudates of wheat (Triticum-aestivum L.) and rye (*Secale cereale* L.)–Possible role in allelopathy. J Chem Ecol. 1991;17: 1037–1043. doi: 10.1007/BF01402932 24259166 10.1007/BF01402932

[3] Poschenrieder C, Torra RP, Barcelo J. A role for cyclic hydroxamates in aluminium resistance in maize. J Inorg Biochem. 2005;99: 1830–1836. doi: 10.1016/j.jinorgbio.2005.05.017 16054220 10.1016/j.jinorgbio.2005.05.017

[4] Schulz M, Marocco A, Tabaglio V, Macias FA, Molinillo JMG. Benzoxazinoids in rye alllopathy- from discovery to application in sustainable weed control and organic farming. J Chem Ecol. 2013;39: 154–174. doi: 10.1007/s10886-013-0235-x 23385365 10.1007/s10886-013-0235-x

[5] Tanwir F, Fredholm M, Gregersen PL, Fomsgaard IS. Comparison of the levels of bioactive benzoxazinoids in different wheat and rye fractions and the transformation of these compounds in homemade foods. Food Chemistry. 2013;141: 444–450. doi: 10.1016/j.foodchem.2013.02.109 23768378 10.1016/j.foodchem.2013.02.109

[6] Brooks AM, Danehower DA, Murphy JP, Reberg-Horton C, Burton JD. Estimation of heritability of benzoxazinoid production in rye (*Secale cereale* L.) using gas chromatographic analysis. Plant Breed. 2012;131: 104–109.

[7] Rakoczy-Trojanowska M, Orczyk W, Krajewski P, Bocianowski J, Stochmal A, Kowalczyk M. ScBx gene based association analysis of hydroxamate content in rye (*Secale cereale* L.). J Appl Genet. 2017a;58: 1–9. doi: 10.1007/s13353-016-0356-3 27465692 10.1007/s13353-016-0356-3 PMC5243912

[8] Butrón A, Chen YC, Rottinghaus GE, Mullen MD Genetic variation at bx controls DIMBOA content in maize. Theor Appl Genet. 2010;120: 721–734. doi: 10.1007/s00122-009-1192-1 19911162 10.1007/s00122-009-1192-1

[9] Zheng LL, McMullen MD, Bauer E, Schon CC, Gierl A, Frey M Prolonged expression of the BX1 signature enzyme is associated with a recombination hotspot in the benzoxazinoid gene cluster in Zea mays. J Exp Botany. 2015;66: 3917–3930. 25969552 10.1093/jxb/erv192 PMC4473990

[10] Rakoczy-Trojanowska M, Krajewski P, Bocianowski J, Schollenberger M, Wakuliński W, Milczarski P, Masojć P, Targońska-Karasek M, Banaszak Z, Brukwiński W, Banaszak K, Orczyk W, Kilian A. Identification of single nucleotide polymorphisms associated with leaf rust resistance, alpha-amylase activity and preharvest sprouting in rye. Plant Mol Biol Rep. 2017b;35(3): 366–378. doi: 10.1007/s11105-017-1030-6 28603340 10.1007/s11105-017-1030-6 PMC5443880

[11] Milczarski P, Bolibok-Brągoszewska H, Myśków B, Stojałowski S, Heller-Uszyńska K, Góralska M, Brągoszewski P, Uszyński P, Kilian A, Rakoczy-Trojanowska M. A high density consensus map of rye (*Secale cereale* L.) based on DArT markers. PloS ONE. 2011;6(12): e28495 doi: 10.1371/journal.pone.0028495 22163026 10.1371/journal.pone.0028495 PMC3232230

[12] Masojć P, Larsson-Raźnikiewicz M. Genetic variation of α-amylase levels among rye (*Secale cereale* L.) kernels, tested by gel diffusion technique. Swedish J Agric Res. 1991;21: 141–145.

[13] Bakera B, Makowska B, Groszyk J, Niziołek M, Orczyk W, Bolibok-Brągoszewska H, Hromada-Judycka A, Rakoczy-Trojanowska M. Structural characteristics of ScBx genes controlling the biosynthesis of hydroxamic acids in rye (*Secale cereale* L.). J Appl Genet. 2015;56: 287–298. doi: 10.1007/s13353-015-0271-z 25666974 10.1007/s13353-015-0271-z PMC4543422

[14] Stam P Construction of integrated genetic linkage maps by means of a new computer package: JoinMap. The Plant Journal. 1993;3: 739–744.

[15] Cullis BR, Smith A, Coombes N. On the design of early generation variety trials with correlated data. JABES. 2006;11: 381–393.

[16] International VSN. GenStat for Windows 16th Edition VSN International, Hemel Hempstead, UK Web page: GenStat.co.uk. 2013.

[17] Wang S, Basten CJ, Zeng ZB. Windows QTL Cartographer 2.5. Department of Statistics, North Carolina State University, Raleigh, NC 2012 (http://statgen.ncsu.edu/qtlcart/WQTLCart.htm)

[18] Sue M, Nakamura C, Nomura T. Dispersed benzoxazinone gene cluster: molecular characterization and chromosomal localization of Glucosyltransferase and Glucosidase Genes in wheat and rye. Plant Physiology. 2011;157: 985–997. doi: 10.1104/pp.111.182378 21875895 10.1104/pp.111.182378 PMC3252142

[19] Nomura T, Ishihara A, Imaishi H, Ohkawa H, Endo TR, Iwamura H. Rearrangement of the genes for the biosynthesis of benzoxazinones in the evolution of Triticeae species. Planta 2003;217: 776–782. doi: 10.1007/s00425-003-1040-5 12734755 10.1007/s00425-003-1040-5

[20] Dutartre L, Hilliou F, Feyereisen R. Phylogenomics of the benzoxazinoid biosynthetic pathway of Poaceae: gene duplication and origin of the Bx cluster. BMC Evolutionary Biology. 2012;12: 64 doi: 10.1186/1471-2148-12-64 22577841 10.1186/1471-2148-12-64 PMC3449204

[21] Devos KM, Atkinson MD, Chinoy CN, Francis HA, Harcourt RL, Koebner RMD, Liu CJ, Masojć P, Xie DX, Gale MD. Chromosomal rearrangements in the rye genome relative to that of wheat. Theor Appl Genet 1993;85: 673–680. doi: 10.1007/BF00225004 24196035 10.1007/BF00225004

[22] Masojć P, Lebiecka K, Milczarski P, Wiśniewska M, Łań A, Owsianicki R. Three classes of loci controlling preharvest sprouting in rye (Secale cereale L.) discerned by means of bidirectional selective genotyping (BSG). Euphytica. 2009;170: 123–129.

[23] Masojć P, Wiśniewska M, Łań A, Milczarski P, Berdzik M, Pędziwiatr D, Pol-Szyszko M, Gałęza M, Owsianicki R. Genomic architecture of alpha-amylase activity in mature rye grain relative to that of preharvest sprouting. J Appl Genet. 2011;52: 153–160. doi: 10.1007/s13353-010-0025-x 21225388 10.1007/s13353-010-0025-x

[24] Masojć P, Bienias A, Berdzik M, Kruszona P. Genetic analysis carried out in population tails reveals diverse two-loci interactions as a basic factor of quantitative traits variation in rye. J Appl Genet. 2016;57: 165 https://doi.org/10.1007/s13353-015-0321-6. doi: 10.1007/s13353-015-0321-6 26450131 10.1007/s13353-015-0321-6 PMC4830850

[25] Masojć P, Banek-Tabor A, Milczarski P, Twardowska M. QTLs for resistance to preharvest sprouting in rye (*Secale cereale* L.). J Appl Genet. 2007;48: 211–217. doi: 10.1007/BF03195215 17666773 10.1007/BF03195215

[26] Masojć P, Milczarski P. Relationship between QTLs for preharvest sprouting and alpha-amylase activity in rye grain. Mol Breed. 2009;23: 75–84.

[27] Milczarski P, Banek-Tabor A, Lebiecka K, Stojałowski S, Myśków B, Masojć P. New genetic map of rye composed of PCR-based molecular markers and its alignment with the reference map of the DS2 × RXL10 intercross. J Appl Genet. 2007;48: 11–24. 17272857 10.1007/BF03194653

[28] Masojć P, Kosmala A. Proteomic analysis of preharvest sprouting in rye using two-dimensional electrophoresis and mass spectrometry. Mol Breed. 2012;30: 1355–1361. doi: 10.1007/s11032-012-9721-z 23024596 10.1007/s11032-012-9721-z PMC3460173

[29] Masojć P, Kosmala A, Perlikowski D. Proteomic analysis of developing rye grain with contrasting resistance to preharvest sprouting. J Appl Genet. 2013;54: 11–19. doi: 10.1007/s13353-012-0127-8 23247911 10.1007/s13353-012-0127-8 PMC3548085

[30] Kato-Noguchi H, Macias FA, Molinillo JMG. Structure-activity relationship of benzoxazinones and related compounds with respect to the growth inhibition and alpha-amylase activity in cress seedlings. J Plant Physiol. 2010;167: 1221–1225. doi: 10.1016/j.jplph.2010.04.006 20605653 10.1016/j.jplph.2010.04.006

[31] Twardowska M, Masojć P, Milczarski P. Pyramiding genes affecting sprouting resistance in rye by means of marker assisted selection. Euphytica. 2005;143: 257–260.

[32] Masojć P, Milczarski P. Mapping QTLs for alpha-amylase activity in rye grain. J Appl Genet. 2005;46: 115–123. 15876678

[33] Myśków B, Stojałowski S, Łań A, Bolibok-Brągoszewska H, Rakoczy Trojanowska M, Kilian A. Detection of the quantitative trait loci for α-amylase activity on a high-density genetic map of rye and comparison of their localization to loci controlling preharvest sprouting and earliness. Mol Breed. 2012;30(1): 367–376. doi: 10.1007/s11032-011-9627-1 22707913 10.1007/s11032-011-9627-1 PMC3362717

[34] Miedaner T, Sperling U. Effect of leaf rust on yield components of winter rye hybrids and assessment of quantitative resistance. J. Phytopathology. 1995;143: 725–730.

[35] Miedaner T, Gey A, Sperling U, Gieger H. Quantitative-genetic analysis of leaf-rust resistance in seedling and adult-plant stages of inbred lines and their testcrosses in winter rye. Plant Breed. 2002;121: 475–479.

[36] Wehling P, Linz A, Hackauf B, Roux SR, Ruge B, Klocke B. Leaf-rust resistance in rye (*Secale cereale* L.) 1. Genetic analysis and mapping of resistance genes Pr1 and Pr2. Theor Appl Genet. 2003;107: 432–438. doi: 10.1007/s00122-003-1263-7 12721636 10.1007/s00122-003-1263-7

