# Supplementary material for: QTL mapping for benzoxazinoid content, preharvest sprouting, α-amylase activity, and leaf rust resistance in rye (Secale cereale L.)
Source: PLoS One. 2017 Dec 21;12(12):e0189912. doi: 10.1371/journal.pone.0189912 (PMC5739458; doi:10.1371/journal.pone.0189912)
Supplement: S1 Protocol — (DOCX) [file pone.0189912.s001.docx]

**QTL mapping for benzoxazinoid content, preharvest sprouting, ⍺-amylase activity, and leaf rust resistance in rye (*Secale cereale* L.)**

Paweł Milczarski^1*^, Piotr Masojć^1^, Paweł Krajewski^2^ Anna Stochmal^3^, Mariusz Kowalczyk^3^ Mihail Angelov^1^, Valentina Ivanova^1^, Małgorzata Schollenberger^4^, Wojciech Wakuliński^4^ Zofia Banaszak^5^, Katarzyna Banaszak^5^, Monika Rakoczy-Trojanowska^4, 6^

^1)^West Pomeranian University of Technology, Szczecin, Poland,

^2)^Institute of Plant Genetics, Polish Academy of Sciences, Poznań, Poland,

^3^)Institute of Soil Science and Plant Cultivation – State Research Institute, Puławy,

^4)^Warsaw University of Life Sciences, Warsaw, Poland;

^5)^Danko Plant Breeders LTD, KOŚCIAN, Poland;

^6)^Polish Academy of Sciences Botanical Garden - Centre For Biological Diversity Conservation in Powsin Warsaw, Poland.

*Corresponding author:

E-mail: [pawel.milczarski@zut.edu.pl](mailto:pawel.milczarski@zut.edu.pl)

**S1 Protocol. Sequences of primers, PCR conditions, and restriction enzymes used for revealing the allelic polymorphisms in SNPs of *ScBx*1-5.**

1. Gene ScBx1

| Marker | Primers | Sequence |
| --- | --- | --- |
| ScBx1 | ScBx1_F1 | ATGTTCTGCCGGTCATTAGG |
|  | ScBx1_R1 | TTTATTTGCCGAGTCATGGA |

The reaction mix was prepared as follows:

| Component (per reaction) | Volume (μl) |
| --- | --- |
| dH_2_O | 4.5 |
| Promega GoTaq G2 Green Master Mix | 7.5 |
| Forward primer (5 pmol) | 1 |
| Reverse primer (5 pmol) | 1 |
| Genomic DNA (20 ng/μl) | 1 |
| Final volume | 15 |

The amplification reaction was performed in a Bio-Rad T100 at the following conditions:

| Step | Temperature (°C) | Time (min:sec) | Number of cycles |
| --- | --- | --- | --- |
| Initial denaturation | 95 | 2:00 | 1 |
| Denaturation | 95 | 0:30 |  |
| Primer annealing | 56 | 1:00 | 30 |
| Elongation | 72 | 1:00 |  |
| Final elongation | 72 | 5:00 | 1 |

The expected size of the products was 1802 bp, which was consistent with the obtained product. This product underwent restriction with HaeIII (NEB), with the following reaction mix and incubation conditions:

| Component (per reaction) | Volume (μl) |
| --- | --- |
| dH_2_O | 19 |
| CutSmart buffer (10x) | 0.5 |
| HaeIII enzyme (10000 U/ml) | 0.5 |
| PCR product | 10 |
| Final volume | 30 |

| Step | Temperature (°C) | Time (min) |
| --- | --- | --- |
| Incubation | 37 | 30 |
| Inactivation | 80 | 20 |


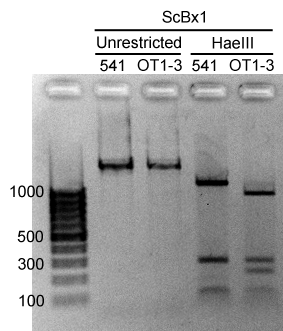


After restriction, the products were separated on a 1.5% agarose gel in 1x TBE buffer at 5 V/cm for 90 minutes, and visualized with Ethidium Bromide staining on an UV trans-illuminator. The size of the attained products was consistent with the size of the products from in-silico restriction analysis.

1. Gene ScBx2

| Marker | Primers | Sequence |
| --- | --- | --- |
| ScBx2 | ScBx2_F1 | GCTCCACGATCTCTCTTGGT |
|  | ScBx2_R1 | ATATGCGCGCACCAAATATC |

The reaction mix was prepared as follows:

| Component (per reaction) | Volume (μl) |
| --- | --- |
| dH_2_O | 4.5 |
| Promega GoTaq G2 Green Master Mix | 7.5 |
| Forward primer (5 pmol) | 1 |
| Reverse primer (5 pmol) | 1 |
| Genomic DNA (20 ng/μl) | 1 |
| Final volume | 15 |

The amplification reaction was performed in a Bio-Rad T100 at the following conditions:

| Step | Temperature (°C) | Time (min:sec) | Number of cycles |
| --- | --- | --- | --- |
| Initial denaturation | 95 | 2:00 | 1 |
| Denaturation | 95 | 0:30 |  |
| Primer annealing | 56 | 1:00 | 30 |
| Elongation | 72 | 1:00 |  |
| Final elongation | 72 | 5:00 | 1 |

The expected size of the products was 1683 bp, which was consistent with the obtained product. This product was restricted with TaqαI (NEB), with the following reaction mix and incubation conditions:

| Component (per reaction) | Volume (μl) |
| --- | --- |
| dH_2_O | 19 |
| CutSmart buffer (10x) | 0.5 |
| TaqαI enzyme (20000 U/ml) | 0.5 |
| PCR product | 10 |
| Final volume | 30 |

| Step | Temperature (°C) | Time (min) |
| --- | --- | --- |
| Incubation | 65 | 30 |
| Inactivation | 80 | 20 |


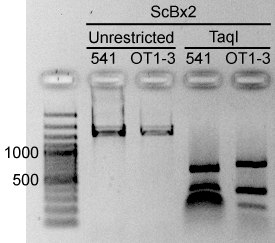
After restriction, the products were separated on a 1.5% agarose gel in 1x TBE buffer at 5 V/cm for 90 minutes, and visualized with Ethidium Bromide staining on an UV trans-illuminator. The size of the attained products was consistent with the size of the products from in-silico restriction analysis.

1. Gene *ScBx*3

| Marker | Primers | Sequence |
| --- | --- | --- |
| ScBx3 | 3358FL | TAATGCACAACTATGACTGTTTCC |
|  | 314RL | TCTCGTGCTCATGTCCAAAT |

The reaction mix was prepared as follows:

| Component (per reaction) | Volume (μl) |
| --- | --- |
| dH_2_O | 4.5 |
| Promega GoTaq Long PCR Master Mix | 7.5 |
| Forward primer (5 pmol) | 1 |
| Reverse primer (5 pmol) | 1 |
| Genomic DNA (20 ng/μl) | 1 |
| Final volume | 15 |

The amplification reaction was performed in a Bio-Rad T100 at the following conditions:

| Step | Temperature (°C) | Time (min:sec) | Number of cycles |
| --- | --- | --- | --- |
| Initial denaturation | 94 | 2:00 | 1 |
| Denaturation | 94 | 0:30 |  |
| Primer annealing | 60 | 5:00 | 35 |
| Elongation | 72 | 1:00 |  |
| Final elongation | 72 | 10:00 | 1 |


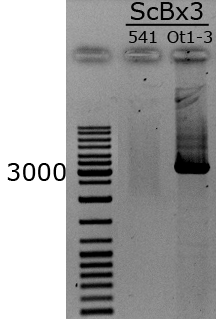
The products of the reaction were separated on a 1% agarose gel in 1x TBE buffer at 5 V/cm for 100 minutes, and visualized with Ethidium Bromide staining on an UV trans-illuminator. As the marker is a dominant one, product was expected only from parental line OT1-3 with size of 3358 bp, which was consistent with the obtained product.

1. Gene ScBx4

| Marker | Primers | Sequence |
| --- | --- | --- |
| ScBx4 | 3066FL | GTTGCATTTCTTTGTAAGTATATA |
|  | 41RL | ATTCGCTGTTACATTTGTAGAGGAT |

The reaction mix was prepared as follows:

| Component (per reaction) | Volume (μl) |
| --- | --- |
| dH_2_O | 4.5 |
| Promega GoTaq Long PCR Master Mix | 7.5 |
| Forward primer (5 pmol) | 1 |
| Reverse primer (5 pmol) | 1 |
| Genomic DNA (20 ng/μl) | 1 |
| Final volume | 15 |

The amplification reaction was performed in a Bio-Rad T100 at the following conditions:

| Step | Temperature (°C) | Time (min:sec) | Number of cycles |
| --- | --- | --- | --- |
| Initial denaturation | 94 | 2:00 | 1 |
| Denaturation | 94 | 0:30 |  |
| Primer annealing | 60 | 3:00 | 35 |
| Elongation | 72 | 1:00 |  |
| Final elongation | 72 | 10:00 | 1 |


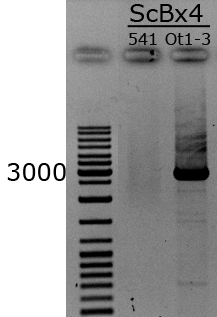
The products of the reaction were separated on a 1% agarose gel in 1x TBE buffer at 5 V/cm for 100 minutes, and visualized with Ethidium Bromide staining on an UV trans-illuminator. As the marker is a dominant one, product was expected only from parental line OT1-3 with size of 3066 bp, which was consistent with the obtained product.

1. Gene ScBx5

| Marker | Primers | Sequence |
| --- | --- | --- |
| ScBx5 | ScBx5_F1 | ACTCATCTGCTTGGCTCGAT |
|  | ScBx5_R1 | CCTCCGTTTGGAATCAACTG |

The reaction mix was prepared as follows:

| Component (per reaction) | Volume (μl) |
| --- | --- |
| dH_2_O | 4.5 |
| Promega GoTaq G2 Green Master Mix | 7.5 |
| Forward primer (5 pmol) | 1 |
| Reverse primer (5 pmol) | 1 |
| Genomic DNA (20 ng/μl) | 1 |
| Final volume | 15 |

The amplification reaction was performed in a Bio-Rad T100 at the following conditions:

| Step | Temperature (°C) | Time (min:sec) | Number of cycles |
| --- | --- | --- | --- |
| Initial denaturation | 95 | 2:00 | 1 |
| Denaturation | 95 | 0:30 |  |
| Primer annealing | 62 | 1:00 | 30 |
| Elongation | 72 | 1:00 |  |
| Final elongation | 72 | 5:00 | 1 |


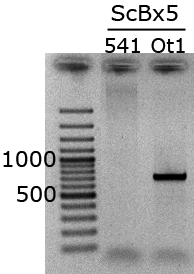


The products of the reaction were separated on a 1.5% agarose gel in 1x TBE buffer at 5 V/cm for 60 minutes, and visualized with Ethidium Bromide staining on an UV trans-illuminator. As the marker is a dominant one, product was expected only from parental line OT1-3 with size of 710 bp, which was consistent with the obtained product.
